# Cyclin T1-Dependent Genes in Activated CD4^+^ T and Macrophage Cell Lines Appear Enriched in HIV-1 Co-Factors

**DOI:** 10.1371/journal.pone.0003146

**Published:** 2008-09-05

**Authors:** Wendong Yu, Rajesh Ramakrishnan, Yan Wang, Karen Chiang, Tzu-Ling Sung, Andrew P. Rice

**Affiliations:** Department of Molecular Virology and Microbiology, Baylor College of Medicine, Houston, Texas, United States of America; National Cancer Institute, United States of America

## Abstract

HIV-1 is dependent upon cellular co-factors to mediate its replication cycle in CD4^+^ T cells and macrophages, the two major cell types infected by the virus in vivo. One critical co-factor is Cyclin T1, a subunit of a general RNA polymerase II elongation factor known as P-TEFb. Cyclin T1 is targeted directly by the viral Tat protein to activate proviral transcription. Cyclin T1 is up-regulated when resting CD4^+^ T cells are activated and during macrophage differentiation or activation, conditions that are also necessary for high levels of HIV-1 replication. Because Cyclin T1 is a subunit of a transcription factor, the up-regulation of Cyclin T1 in these cells results in the induction of cellular genes, some of which might be HIV-1 co-factors. Using shRNA depletions of Cyclin T1 and transcriptional profiling, we identified 54 cellular mRNAs that appear to be Cyclin T1-dependent for their induction in activated CD4^+^ T Jurkat T cells and during differentiation and activation of MM6 cells, a human monocytic cell line. The promoters for these Cyclin T1-dependent genes (CTDGs) are over-represented in two transcription factor binding sites, SREBP1 and ARP1. Notably, 10 of these CTDGs have been reported to be involved in HIV-1 replication, a significant over-representation of such genes when compared to randomly generated lists of 54 genes (p value<0.00021). The results of siRNA depletion and dominant-negative protein experiments with two CTDGs identified here, CDK11 and Casein kinase 1 gamma 1, suggest that these genes are involved either directly or indirectly in HIV-1 replication. It is likely that the 54 CTDGs identified here include novel HIV-1 co-factors. The presence of CTDGs in the protein space that was available for HIV-1 to sample during its evolution and acquisition of Tat function may provide an explanation for why CTDGs are enriched in viral co-factors.

## Introduction

The major targets of HIV-1 infection are CD4^+^ T cells and macrophages, as these two cell types express CD4 and CCR5 or CXCR4, the receptor and co-receptor, respectively, for the viral Envelope protein. However, the ability of CD4^+^ T cells and macrophages to support a productive HIV-1 infection depends upon the physiological status of the cell. Fully resting CD4^+^ T cells are non-permissive for HIV-1 replication and must be activated to support high levels of viral replication [1], [2]. Monocytes are also non-permissive for replication and must undergo a program of macrophage differentiation to support HIV-1 infection [3]. In resting CD4^+^ T cells and monocytes, viral entry is not limiting for infection, but rather a number of post-entry steps in the replication cycle are defective, including: reverse transcription [1], [4], [5]; nuclear import and integration of the viral cDNA [6], [7]; nuclear export of spliced viral mRNAs [8]; RNA polymerase II transcription of the integrated provirus [9]. These defects in viral replication are likely, in part, to be the result of limiting levels of cellular co-factors that mediate the various steps in the viral life cycle. By expressing adequate levels of all essential cellular co-factors, activated CD4^+^ T cells and differentiated macrophages provide an optimal environment for HIV-1 replication.

One essential co-factor for HIV-1 replication is Cyclin T1 which is targeted by the viral Tat protein to mediate RNA polymerase II transcription of the integrated provirus (reviewed in [10]–[13]). Cyclin T1 is one of several regulatory subunits of a set of general elongation factors known collectively as P-TEFb [14], [15]. The transcriptional elongation of most cellular genes is stimulated by P-TEFb [16], [17]. All P-TEFb complexes contain CDK9 as the catalytic subunit, either the major 42 kDa protein or a minor 55 kDa protein that arises from an upstream transcriptional start site [18]. P-TEFb complexes differ according to their regulatory subunit, either Cyclin T1, two isoforms of Cyclin T2 termed Cyclin T2a and T2b, or Cyclin K. A substantial portion of P-TEFb is in a catalytically repressed form because of its association with 7SK snRNA and the HEXIM1 or HEXIM2 proteins [19]–[22]. The association of P-TEFb with 7SK snRNA and HEXIM proteins may sequester P-TEFb in a complex from which functional P-TEFb can be rapidly recruited to regulate RNA polymerase II transcription [14], [15].

The HIV-1 Tat protein specifically targets the Cyclin T1/P-TEFb complex through a direct protein-protein interaction with Cyclin T1 [23]. In the absence of the viral Tat protein, RNA polymerase II initiates transcription within the viral 5′ long terminal repeat sequences, but the polymerase is non-processive due to the action of two negative factors, DSIF and NELF, that limit elongation [11]. After expression of the Tat protein, Tat binds to Cyclin T1/P-TEFb, and this protein complex then binds to the TAR RNA element that forms at the 5′ end of nascent viral transcripts. The CDK9 subunit of P-TEFb can then phosphorylate the carboxyl terminal domain of the large subunit of RNA polymerase II and DSIF and NELF, thereby converting RNA polymerase to a processive enzyme that is able to transcribe the entire proviral genome.

Cyclin T1 protein expression is highly regulated in CD4^+^ T cells and macrophages, and this regulation correlates with permissibility for HIV-1 replication. Cyclin T1 expression is low in resting CD4^+^ T cells isolated from healthy donors, but upon T cell activation, it is induced by a mechanism that appears to involve post-transcriptional regulation [24], [25]. Cyclin T1 expression is also low in freshly isolated monocytes, and it is up-regulated by a post-transcriptional mechanism within one to two days after the cells are cultured under conditions that allow macrophage differentiation [26]. However, after one to two weeks in culture, Cyclin T1 protein expression is shut-off in macrophages by proteasome-mediated proteolysis. Treatment of macrophages with the immunosuppressive cytokine IL-10 can accelerate this proteasome-mediated shut-off of Cyclin T1 [27]. After it is shut-off in late-differentiated macrophages, Cyclin T1 can be re-induced by activation with agents such as LPS or by HIV-1 infection, indicating that the induction of Cyclin T1 is a component of an innate immune response in mature macrophages [28], [29]. In contrast to the regulated expression of Cyclin T1, the Cyclin T2 regulatory subunit of P-TEFb is expressed constitutively in resting CD4^+^ T cells and is not further induced by T cell activation [24]. Cyclin T2 is also expressed constitutively throughout macrophage differentiation and it is not induced by macrophage activation [29].

Given its role as the regulatory subunit of a general RNA polymerase II elongation factor, the up-regulation of Cyclin T1 during T cell activation and macrophage differentiation or activation suggests that Cyclin T1 is likely to be involved in the induced transcription of many cellular genes in these cells. Our recent study utilized shRNA depletions and DNA microarrays to examine Cyclin T1-dependent genes (termed here CTDGs) in MM6 cells, a monocytic cell line that can be induced to differentiate into a macrophage-like phenotype by PMA treatment [30]. We found that like primary monocytes, Cyclin T1 is expressed at a low level in MM6 cells and it is up-regulated by a post-transcriptional mechanism when differentiation is induced by PMA. Also like primary monocytes/macrophages, the Cyclin T2 subunit of P-TEFb is expressed constitutively in MM6 cells and is not up-regulated by PMA treatment. When Cyclin T1 expression was depleted by shRNA, the expression of more than 20% of PMA-induced genes in MM6 cells was inhibited, indicating that the up-regulation of Cyclin T1 during monocyte differentiation is required for the expression of a large portion of differentiation-induced genes.

Our previous study identified CTDGs in PMA-treated MM6 cells, a setting that models the permissive environment for HIV-1 infection in differentiated macrophages [30]. In the present study, we used shRNA depletions and DNA microarrays to identify CTDGs in LPS-activated MM6 cells, another environment that can be permissive for HIV-1 infection [31]. We also identified CTDGs in activated Jurkat CD4^+^ T cells, an additional environment that is permissive for HIV-1 replication. We used the DNA microarray data to identify a common set of 54 CTDGs that are induced in activated CD4^+^ T cell and differentiated and activated macrophages. The promoters for these genes are of interest, as their regulation is similar to that of the HIV-1 LTR: Cyclin T1-dependent and inducible by T cell activation and macrophage differentiation and activation. Remarkably, this set of CTDGs is highly over-represented in proteins with a reported role in HIV-1 replication. Our analysis of two genes in the list, CDK11 and Casein kinase1 gamma 1 (CSNK1G1), suggests that these two genes also play a role in HIV-1 replication. It is therefore likely that the list of CTDGs identified here contains novel HIV-1 co-factors.

## Results

### Depletion of Cyclin T1 in MM6 and Jurkat T cells

To identify Cyclin T1-dependent genes (CTDGs), we used a shRNA vector to deplete Cyclin T1 and evaluated the effects on global gene expression with DNA microarrays. Genetic manipulation of primary macrophages and CD4^+^ T cells by shRNA methodologies poses considerable technical challenges. Additionally, variability between cells from different donors introduces a complicating factor. Because of these technical issues, we carried out Cyclin T1-depletion experiments in transformed cell lines. As a model for activated CD4^+^ T cells, we used Jurkat T cells, a cell line derived from a human leukemia that has been widely used in T cell activation studies [32], [33]. As a model for monocytes/macrophages, we used the Mono-Mac 6 (MM6) cell line. This cell line is from a human leukemia and it exhibits characteristics of mature monocytes, such as the expression of markers specific for mature monocytes which are absent in the less mature human promonocytic cells lines U937 and THP1 [34]. MM6 cells can be induced to differentiate to a macrophage-like phenotype by PMA, and they can be activated by LPS.

The shRNA lentiviral vector that we used expresses an eGFP marker protein and does not express any lentiviral gene products [35]. We constructed a lentiviral vector, termed shRNA-T1, which expresses a shRNA that targets Cyclin T1 mRNA. We also constructed a negative control shRNA vector, termed shRNA-Con, which expresses a four-nucleotide mismatch to Cyclin T1 mRNA. We previously used these lentiviral vectors and DNA microarrays to identify CTDGs in PMA-treated MM6 cells [30].

**Figure 1:**
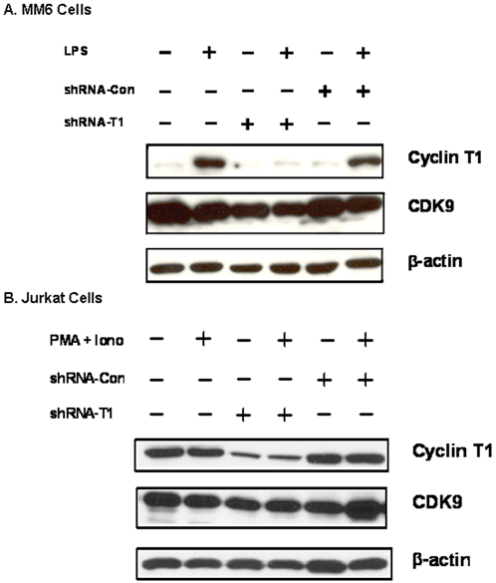
A) MM6 cells: non-infected or MM6 cells infected at an m.o.i. of five with the indicated lentiviral vector were cultured for five days. Cells were then treated with LPS for 24 hours as indicated. Cell extracts were prepared and immunoblots performed to measure levels of Cyclin T1, CDK9, and β-actin. B) Jurkat cells: non-infected or Jurkat cells infected at an m.o.i. of five with the indicated lentiviral vector were cultured for five days. Cells were then treated with PMA+ ionomycin for 24 hours as indicated. Cell extracts were prepared to measure expression of Cyclin T1, CDK9, and β-actin.

Without LPS activation, the basal level of expression of Cyclin T1 in MM6 cells is low (Fig. 1A). LPS activation resulted in a strong induction of Cyclin T1 in both parental MM6 cells and cells transduced with the shRNA-Con vector. In contrast, Cyclin T1 expression was not induced in LPS-activated cells that were transduced with the shRNA-T1 vector. Flow cytometry analysis showed that >90% of cells in all transduced cultures expressed the eGFP marker protein (data not shown). We observed in real-time RT-PCR assays that Cyclin T1 mRNA levels were reduced approximately four-fold in cells infected with the shRNA-T1 vector (data not shown).

Unlike MM6 cells, the basal level expression of Cyclin T1 in Jurkat cells is high and it is not further induced by activation with PMA+ ionomycin (Fig. 1B). We have observed a similar high basal expression level of Cyclin T1 in the CD4^+^ T cell lines CEM and H9, and this basal level is not further induced by activation (unpublished results). Cyclin T1 expression was not affected in Jurkat cells transduced with the shRNA-Con vector. In contrast, Cyclin T1 expression was significantly reduced in untreated and activated Jurkat cells that were transduced with the shRNA-T1 vector. Flow cytometry analysis indicated that >90% of cells in all transduced cultures expressed the eGFP marker protein (data not shown). Using real-time RT-PCR assays, we also observed that the mRNA levels of Cyclin T1 were reduced approximately four-fold in shRNA-T1 transduced cells relative to shRNA-Con transduced cells and parental Jurkat cells (data not shown). The data presented in Figure 1 demonstrates that the shRNA-T1 vector is effective in depleting Cyclin T1 protein expression and this system can be used to identify CTDGs in LPS-treated MM6 cells and PMA/ionomycin-treated Jurkat cells.

### Transcriptional profiling: validation and analysis of microarray data

To examine how the depletion of Cyclin T1 affects global gene expression, we utilized Affymetrix human genome U133 Plus 2.0 DNA arrays representing about 18,953 unique (non-redundant) transcripts. DNA microarrays were used to analyze two independent biological replicate experiments of MM6 cells treated with LPS and Jurkat cells treated with PMA/ionomycin. DNA microarrays were also used to analyze three biological replicates with PMA treated MM6 cells. To verify that the microarray data are reliable, we performed real-time PCR assays for several mRNAs whose levels in microarrays were affected by the various treatments. For all mRNAs assayed, the PCR-based assays were in accordance with the microarray data, indicating that the microarray data are in general reliable (Fig. S1). We performed a similar PCR assay validation of the microarray data in Cyclin T1-depletions of PMA-treated MM6 cells [30]. Additionally, dendograms were generated based upon the expression measures of all probes sets on the Affymetrix array (Figs. S2, S3). These dendrograms demonstrated that RNA preparations from cells subjected to the various treatments partitioned into the expected groups, further indicating that the microarray data are reliable. The primary microarray data was deposited in the NCBI GEO database (see Materials and Methods).

### Cellular genes repressed by Cyclin T1 depletions in both activated MM6 and Jurkat cells

**Figure 2:**
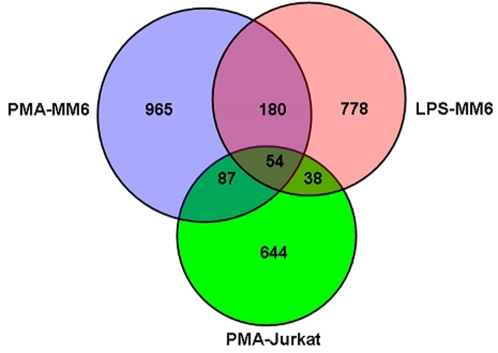
CTDGs. The Venn diagram represents the number of genes whose mRNAs were reduced >1.5-fold (p value<0.05) by Cyclin T1 depletions. Cyclin T1-dependency was observed for 965 of the PMA-inducible genes in MM6 cells, 778 of the LPS-inducible genes in MM6 cells, and 644 of the PMA/ionomycin-inducible genes in Jurkat cells. The number of genes in intersections of gene sets is indicated.

We used the transcriptional profiling data to identify the genes that were repressed >1.5-fold (p-value<0.05) by Cyclin T1 depletions relative to the shRNA-Con vector in PMA/ionomycin-treated Jurkat cells, PMA-treated MM6 cells, and LPS-treated MM6 cells. There were 644 such genes in Jurkat cells, 965 in PMA-treated MM6 cells and 778 in LPS-treated MM6 cells (Fig. 2). The intersection of these three gene sets contain the 54 genes listed in Table 1. We performed a gene ontology analysis to determine if this gene set was over-represented in any biological processes. A number of processes involved in intracellular transport and localizations of proteins were found to be over-represented (Fig. S4). Six genes in the list are related to these biological processes: Adaptor-related protein complex 1, mu 1 subunit (AP1M1); FYN binding protein (FYB-120/130); Pituitary tumor-transforming 1 interacting protein (PTTG1IP); RAB1A; RAB7L1; VPS24 (also called CHMP3). Given this over-representation of genes involved in protein transport and localization, it is intriguing that these processes are involved in HIV-1 Gag trafficking and virion budding [36], [37].

**Table 1:** 

Gene Name (Gene ID)	Gene Name (Gene ID)
1. Golgi autoantigen, golgi subfamily a, 7 (GOLGA7)	28. Glycolipid transfer protein (GLTP)
2. Nucleosome assembly protein 1-like 5 (NAP1L5)	29. Rho GTPase activating protein 30 (ARHGAP30)
3. NSFL1 (p97) cofactor (p47) (NSFL1C)	30. Pyruvate dehydrogenase kinase, isozyme 3 (PDK3)
***4. S-phase kinase-associated protein 2 (p45) (SKP2)***	31. Chromosome X open reading frame 40A (Cxorf40A)
5. ATPase, Class VI, type 11A (ATP11A)	32. Niemann-Pick disease, type C2 (NPC2)
***6. Adaptor-related protein complex 1, mu 1 subunit (AP1M1)***	33. Iduronate 2-sulfatase (Hunter syndrome) (IDS)
***7. Deoxyhypusine synthase (DHPS)***	34. Eukaryotic translation initiation factor 2C, 4 (EIF2C4)
8. Casein kinase 1, gamma 1 (CSNK1G1)	35. Pituitary tumor-transforming 1 interacting protein (PTTG1IP)
9. Hypothetical protein LOC144438 (LOC144438)	36. Adenosine deaminase, RNA-specific, B1 (RED1 homolog rat) (ADARB1)
***10. MHC, class I-related (MR1)***	37. KIAA1458 protein (KIAA1458)
11. RAB7, member RAS oncogene family-like 1 (RAB7L1)	38. Disabled homolog 2, mitogen-responsive phosphoprotein (Drosophila) (DAB2)
***12. Actin related protein 2/3 complex, subunit 1A, 41kDa (ARPC1A)***	39. Potassium large conductance calcium-activated channel, subfamily M, beta member 1 (KCNMB1)
13. Lipase A, lysosomal acid, cholesterol esterase (LIPA)	40. CTTNBP2 N-terminal like (CTTNBP2NL)
14. Coiled-coil domain containing 32 (CCD32)	41. Neuroblastoma RAS viral (v-ras) homolog (NRAS)
15. Dihydropyrimidinase-like 2 (DPYSL2)	***42. Lysosomal-associated membrane protein 1 (LAMP1)***
16. CDK11: Cell division cycle 2-like 2 (PITSLRE proteins) (CDCL2L2)	43. Chromosome 1 open reading frame 121 (C1orf21)
***17. Caspase recruitment domain family, member 6 (CARD6)***	44. Transmembrane protein 107 (TMEM107)
18. Cytochrome b reductase 1 (CYBRD1)	***45. Granulin (GRN)***
19. Phosphatase and actin regulator 4 (PHACTR4)	46. Cathepsin B (CTSB)
20. 1-acylglycerol-3-phosphate O-acyltransferase 3 (AGPAT3)	47. Transmembrane protein 106A (TMEM106A)
21. Pyridoxal (pyridoxine, vitamin B6) kinase (PDXK)	48. LSM12 homolog (S. cerevisiae) (LSM12)
22. MYC associated factor X (MAX)	***49. Ankyrin repeat domain 6 (ANKRD6)***
23. Tubulin, beta 2B (TUBB2B)	***50. Vacuolar protein sorting 24 homolog (S. cerevisiae) (VPS24)***
24. RAB1A, member RAS oncogene family (RAB1A)	51. Hepatitis A virus cellular receptor 2 (HAVCR2)
25. DEAD box polypeptide 18 (DDX18)	52. Ras association (RalGDS/AF-6) domain family 2 (RASSF2)
26. DAZ associated protein 2 (DAZAP2)	53. Integrin beta 4 binding protein (ITGB4BP)
27. FYN binding protein (FYB-120/130) (FYB)	**54.** Membrane protein, palmitoylated 5 (MAGUK p55 subfamily member 5) (MPP5)

### SEBP1 and ARP1 binding sites are over-represented in CTDGs

The CTDGs listed in Table 1 are Cyclin T1-dependent and inducible by T cell activation, macrophage differentiation, and macrophage activation. Because this regulation is similar to that of the HIV-1 LTR subjected to the same treatments in these cell types, we were interested in identifying transcription factor binding sites in the promoters of the CTDGs that are over-represented relative to the expected number in 54 random promoters. We therefore used the TOUCAN program [38] to examine transcription factor binding (TFBS) sites. We extracted 1.5 kb upstream of the first exon of the 54 genes, as it has been estimated that approximately 75% of human core promoters lie within 1,500 bp of the transcription start site [39]. Using a database of transcription factors and their binding sites TRANSFAC (7.0 public, Vertebrates), TFBS in the promoters were predicted against a background model obtained from Eukaryotic Promoter Database. TFBSs that were statistically over-represented were identified by comparison of the frequency of each transcription factor in our data set against their background frequency.

**Figure 3:**
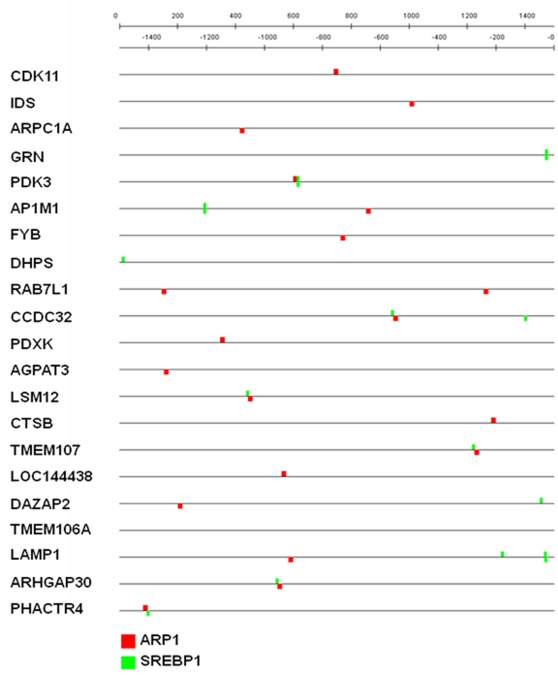
SREBP1 binding sites in CTDGs. The 1.5 kb sequences upstream of the first exon of genes are indicated (both strands). Predicted binding sites on both DNA strands are indicated.

We found that binding sites for two transcription factors, SREBP1 and ARP1 (also called COUP-TFII or NR2F2), were over-represented in the promoters for the 54 genes. As shown in Figure 3, a total of 17 SREBP1 binding sites and 19 ARP1 binding sites were found in the set of 54 promoters. Both SREBP1 and ARP1 are over-represented over their expected frequencies, with p values<0.00013 and <0.0003, respectively. SREBP1 (sterol regulatory element binding transcription factor 1) positively regulates genes involved in fatty acid and cholesterol synthesis [40]. ARP1 is involved in the regulation of genes involved in metabolism of glucose, cholesterol and xenobiotics [41]. No other transcription factor binding sites were found to be statistically over-represented, including those for Sp1, NF-κB, and TFIID. These are notable absences as the HIV-1 LTR contains two highly conserved NF-κB sites, three highly conserved Sp1 binding sites, and an essential TATA element.

### CTDGs are enriched in factors involved in HIV-1 replication

We examined our list of CTDGs for published links to HIV-1 replication. Notably, 10 proteins were found to have a link to HIV-1 (indicated by bold italics in Table 1): 1) SKP2 is involved in Tat function through the ubiquitylation of the CDK9 subunit of P-TEFb [42]; 2) AP1m1 is utilized by the HIV-1 Nef protein to down-regulate CD4 and MHC I [43], [44]; 3) deoxyhypusine synthase is involved in Rev function through an unusual protein modification of its co-factor eIF-5A [45], [46]; 4) Actin related protein 2/3 complex, subunit 1A (ARPC1A) is involved in an HIV-1 pre-integration step and in viral budding [47], [48]; 5) CARD6 is involved in NF-κB activation [49]; 6) LAMP1 co-localizes with the HIV-1 Gag protein in macrophages [50]; 7) granulin is involved in Tat function through an association with Cyclin T1 [51]; 8) VPS24 (CHMP3) is involved in viral budding [52]; 9,10) MHC class I-related (MR1) and Ankyrin repeat domain 6 (ANKRD6) act prior to viral budding [48].

After finding that 10 genes in our set of 54 have a link to HIV-1 replication, we were interested to determine if this number is significantly greater than in sets of 54 genes chosen at random. To generate legitimate random lists, we selected only genes present in the Affymetrix microarrays that were expressed at significant levels in both MM6 cells and Jurkat T cells (see Materials and Methods). We generated seven sets of 54 random genes and analyzed each for published links with HIV-1 replication. We found that these sets had from one to four genes (average 2.4) with a reported link to HIV. Thus, the gene set shown in Table 1 is significantly over-represented in links to HIV-1 replication relative to randomly generated sets of 54 genes (p value<0.00021). This finding indicates that the CTDGs listed in Table 1 are highly likely to contain novel HIV-1 co-factors.

### CDK11 depletion affects Tat function

We investigated two genes in our 54 gene list for a role in HIV-1 replication. Our attention was drawn to CDK11, as an RNAi screen in *Drosophila* observed that CDK11 and CDK9 are linked in a genetic network that regulates the Hedgehog pathway [53]. Because CDK9 is a component of the Tat co-factor P-TEFb, we asked whether a genetic link between these two kinases exists in Tat function.

CDK11 is a ubiquitously expressed member of the CDK family. There is considerable complexity in human CDK11, as it is encoded in two distinct but highly similar genes that arose by gene duplication – *Cdc2L1* and *Cdc2L2 (Cell division control 2 Like)*. Differential splicing from these two genes generate more than 20 distinct CDK11 mRNAs that encode two related proteins, CDK11^p110^ and CDK11^p58^. During apoptosis, a third CDK11 p46 isoform is generated by caspase cleavage of the p110 and p58 proteins. The available data indicate that the p110, p58, and p46 CDK11 proteins are involved in mRNA production, mitosis, and apoptosis, respectively [54].

**Figure 4:**
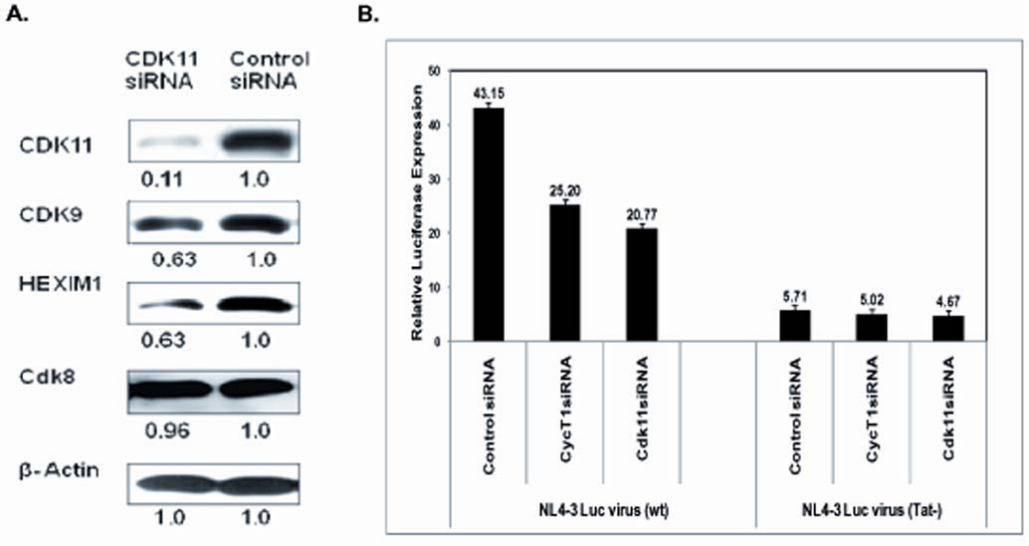
CDK11 depletion reduces protein expression of CDK9 and HEXIM1 and Tat transactivation of HIV-1 provirus. A. HeLa cells were transfected with siRNAs against CDK11 siRNA or control siRNAs. Cells were harvested 72 h post-transfection and cell lysates were analyzed for expression of CDK11, CDK9, HEXIM1, and CDK8 by immunoblots. B. HeLa cells were infected with a lentiviral DHFR-Renilla Luciferase virus with GFP marker. Cells sorted for GFP were then infected with either HIV-1 NL4-3-firefly Luciferase virus (wild type) or with HIV-1 NL4-3 firefly Luciferase virus encoding a mutant Tat (Tat−) for 48 h. Cultures were transfected with the indicated siRNAs. Cell extracts were prepared 48 h post-transfection and assayed for Luciferase expression. Results are presented as the ratio of firefly to Renilla Luciferase expression.

To deplete CDK11 globally, we used siRNAs that target a conserved RNA sequence present in all transcripts from the two CDK11 genes. As shown in Figure 4A, these siRNAs are effective in depleting the CDK11 p110 protein, the isoform involved in mRNA production. The CDK11 depletion also resulted in an approximate 40% decrease in expression of CDK9 and HEXIM1, but not β-actin, CDK8, and CDK7 (not shown). The reduction in CDK9 and HEXIM1, although relatively modest, is specific and reproducible and suggests that CDK11 may somehow regulate the expression of these proteins. The effect of the depletion on HEXIM1 is quite notable, as about 50% of P-TEFb in HeLa cells is associated in a complex with HEXIM1 (or the minor HEXIM2) protein and a small nuclear RNA known as 7SK snRNA. P-TEFb is catalytically inactive when associated with HEXIM1/7SK. The association of P-TEFb with HEXIM1/7SK may sequester excess P-TEFb in a complex from which functional P-TEFb can be rapidly recruited to regulated RNA polymerase II transcription [14], [15]. Using propidium iodide staining and flow cytometry, we observed that there is no apoptosis or cytotoxicity resulting from the CDK11-depletion prior to 72 hr post-transfection with the CDK11 siRNAs. Thus, the two-fold reduction in CDK9 and HEXIM1 protein levels is unlikely to be the result of cytotoxicity or apoptosis. We also observed that shRNA depletions of CDK9 did not affect the expression level of CDK11 (data not shown), suggesting that CDK9 may be downstream of CDK11.

We examined whether the CDK11-depletion had an effect on expression of an HIV-1 Luciferase reporter virus. This virus contains Luciferase in place of the Nef gene and contains a deletion of Envelope; it is pseudotyped with the VSV G glycoprotein to allow entry. We constructed a control HIV-1 Luciferase reporter virus that has an inactive Tat protein; Luciferase expression from this reporter virus is therefore largely Tat-independent [27]. A pool of HeLa cells engineered to express Renilla Luciferase from the DHFR promoter (an internal control that is P-TEFb-independent [30]) was infected with either the Tat^+^ or Tat^−^ Luciferase reporter virus. At 48 hours post-infection with reporter viruses, siRNAs against CDK11, negative control siRNAs, or a positive control of siRNAs against Cyclin T1 were transfected into cells. Cell extracts were then prepared 48 hours after siRNA transfections and Luciferase expression was measured.

As shown in Figure 4B, Luciferase expression from the Tat+ virus was reduced ∼40% by siRNAs against Cyclin T1 relative to the control siRNAs. The siRNAs against CDK11 reduced expression ∼50% relative to the control siRNAs, a slightly more severe reduction than the Cyclin T1 siRNAs. Relative to the control, Luciferase expression from the Tat- virus was reduced only ∼10% and ∼20% by siRNAs against Cyclin T1 and CDK11, respectively. These data indicate that Tat function is especially sensitive to depletion of either Cyclin T1 or CDK11. Thus, like the Hedgehog pathway in *Drosophila*, CDK9 (P-TEFb) and CDK11 appear to be functionally linked for Tat function. However, it is possible that CDK11 is linked rather indirectly to Tat function through effects on CDK9 expression levels or the processing of HIV-1 transcripts.

### Casein kinase 1gamma1 (CSNK1G1) reduces virion infectivity

One of the genes in our gene set, Casein kinase 1 gamma (CSNK1G1), is a plasma membrane-bound member of the casein kinase 1 (CK1) family. Although CSNK1G1 has not been extensively studied, the *Xenopus* protein has been found to be involved in Wnt signaling [55]. Given the intriguing localization of this kinase at the plasma membrane, the site of retroviral assembly and budding, we carried out a preliminary analysis of the role of CSNK1G1 in HIV-1 replication.

**Figure 5:**
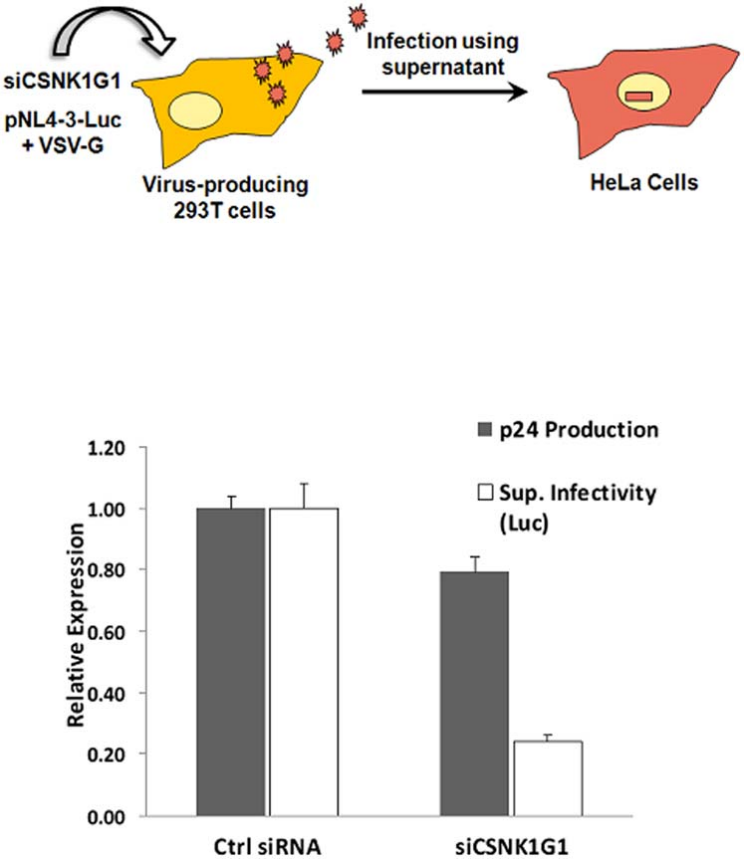
CSNK1G1 depletions. As illustrated, 293T cells were transfected with either control siRNAs or siRNAs against CSNK1G1. One day later, cells were co-transfected with the pNL4-3-Luc and pVSV-G plasmids for virus production. Supernatants were collected 48 hours later and p24 levels were measured by an ELISA assay. Infectivity of supernatants containing reporter the NL-4-3-Luc reporter viruses were measured by infecting HeLa cells with equal volume of supernatants, and Luciferase expression was measured 48 hours post-infection. Luciferase expression was normalized to the amount of p24 in the input supernatants.

In an experimental strategy illustrated in Figure 5, cultures of 293T cells were transfected with siRNAs against CSNK1G1 (or control siRNAs), and then transfected with a HIV-1 pNL4-3-Luc proviral plasmid (deleted for Envelope) and a plasmid that expresses the VSV G glycoprotein to allow production of infectious pseudotyped viruses. This HIV-1 provirus contains Luciferase in place of Nef. Culture supernatants were collected 48 hours post-transfection with the proviral and VSV G plasmids. The amount of p24 in culture supernatants was reduced ∼20% in the CSKN1G1-depleted cells relative to the control siRNA cells (Fig. 5). Equal volumes of the supernatants from 293T cells containing the HIV-1 reporter viruses were used to infect HeLa cells, and Luciferase expression was measured 48 hours later. Luciferase expression was then normalized to the amount of p24 in the 293T supernatant inoculum (Fig. 5). The data indicate that the infectivity of viruses produced in the CSNK1G1-depleted cells was reduced five-fold relative to that produced in the control siRNA cells.

**Figure 6:**
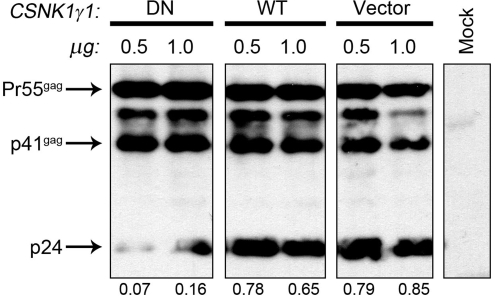
Effects of over-expression of wild type and dn-CSNK1G1 proteins on Gag processing. Cultures of 293T cells were transfected with 0.5 or 1.0 µg wild type CSNK1G1, dn-CSNK1G1, or parental vector (pcDNA 3.0) expression plasmids. One day later, cultures were transfected with pNL4-3-GFP proviral reporter plasmid and cell extracts were prepared one day later. Gag processing in virions associated with cells were examined in an immunoblot using a p24 monoclonal antibody. The ratio of p24 to the 55 kDa Gag precursor is indicated at the bottom of the immunoblot and it was and it was quantified by scanning the film and software analysis.

To begin to explore mechanisms whereby CSNK1G1 may be involved in virion infectivity, we examined the effects on Gag processing of the over-expression of a dominant-negative (dn) CSNK1G1 protein. The dn-CSNK1G1 contains a point mutation (K73R) in the ATPase domain that inactivates catalytic function [55]. Cultures of 293T cells were transfected with 0.5 or 1.0 µg of expression plasmids for wild type CSNK1G1, dn-CSNK1G1, or parental vector and one day later cultures were transfected with a pNL4.3-GFP proviral reporter plasmid. One day after transfection of the proviral reporter plasmid, cell lysates were prepared and processing of cell-associated Gag was examined in an immunoblot (Fig. 6). In cultures transfected with the dn-CSNK1G1 plasmids, processing of Gag to p24 as quantified by the ratio of p24 to the 55 kDa Gag precursor was significantly reduced relative to cultures co-transfected with the wild type CSNK1G1 plasmid or parental vector. This observation suggests that perturbation of CSKN1G1 function by the dominant negative protein can inhibit Gag processing. The data shown in Figures 5 and 6 indicate that CSKN1G1 appears to be a co-factor involved in virion infectivity.

## Discussion

### CTDGs are likely to contain novel HIV-1 co-factors

Using a shRNA vector to deplete Cyclin T1, we identified a common set of 54 Cyclin T1-dependent genes (CTDGs) in PMA/ionomycin-activated Jurkat T cells, PMA-treated MM6 cells, and LPS-treated MM6 cells. Because Cyclin T1 is a general RNA polymerase II transcription factor, its depletion will affect genes that are directly regulated by Cyclin T1. The expression levels of some additional mRNAs are likely to be dependent upon genes that are direct targets of Cyclin T1. Our shRNA depletion of Cyclin T1 therefore likely resulted in down-regulation of some mRNAs through indirect mechanisms. Additionally, as we used a single shRNA against Cyclin T1 in this study, we cannot exclude the possibility that some of the CTDGs identified here are the results of off-target effects of the shRNA. Nevertheless, the CTDGs are highly enriched in SEBP1 and ARP1 binding sites in their promoters (p values<0.00013 and <0.0003) and in genes related to HIV-1 replication (p value<0.00021), strongly suggesting that our CTDG gene set has biological relevance. Until further characterization, it will remain possible that some of these 54 genes are false-positives for their dependency on Cyclin T1.

The 54 CTDGs identified in this study include 10 genes with a reported link to HIV-1 replication: ARPC1A acts at a pre-integration step and at viral budding [47], [48]; CARD6 is involved in NF-κB activation [56]; SKP2 and granulin are involved in Tat function [57], [58]; deoyhypusine synthase is involved in Rev function [59]; AP1M1 is involved in Nef function [60], [61]; LAMP-1 appears to be involved in Gag function [50]; VPS24 is involved in viral budding [62]; ANKRD6 and MR1 act prior to viral budding [48]. As discussed in Results, the identification of 10 of 54 genes with a link to HIV-1 replication is a highly significant over-representation (p value<0.00021) over the expected number (2.4) in randomly generated lists of 54 genes that are expressed in both Jurkat and MM6 cells. It is probable that future genome wide siRNA screens will identify additional genes involved in HIV-1 replication. If this is the case, random lists of 54 genes are likely to contain by chance more than 2.4 genes involved in HIV-1 replication. However, if these new co-factors are identified, it is possible that they will include some of the CTDGs identified here and our gene list may retain its over-representation of genes linked to HIV-1 replication.

An additional CTDG in our list is probably linked to HIV-1 replication – RAB1A (Table 1, gene 24) interacts with RUSC2 which was identified as an HIV-1 co-factor in a recent large scale siRNA screen [48]. We have also provided evidence here that both CDK11 and CSKN1G1 are linked to HIV-1 replication. We conclude that this over-representation of genes with relevance to HIV-1 strongly suggests that our list of CTDGs contains a number of novel HIV-1 co-factors. Future studies using siRNA depletions will determine which of the CTDGs are important in the HIV-1 life cycle.

### Why are CTDGs enriched in HIV-1 co-factors?

During its course of evolution into an infectious agent, HIV-1 acquired Cyclin T1 to mediate Tat stimulation of RNA polymerase II elongation of the provirus. Cyclin T1 is a general transcription factor, and the induced expression of Cyclin T1 in activated CD4^+^ T cells and macrophage drives the expression of many cellular genes. The protein products of many of these CTDGs were therefore always present in infected cells as the virus acquired Cyclin T1 as a co-factor and optimized its replication cycle during evolution. This enrichment of CTDGs in the protein space that was available for HIV-1 to sample during its evolution in CD4+ T cells and macrophages may explain why our list of 54 CTDGs is over-represented in viral co-factors. Our method to identify CTDGs in CD4+ T cells and macrophages was not exhaustive and it seems likely that a number of additional CTDGs serve as viral co-factors.

### Binding sites for SREBP1 and ARP1 are enriched in promoters for CTDGs

The HIV-1 LTR and the CTDGs identified here have similar patterns of regulation: Cyclin T1-dependent and inducible by T cell activation and macrophage differentiation or activation. Because the HIV-1 LTR contains two conserved NF-κB sites, three conserved Sp1 sites, and a conserved TATA element [63], it is notable that binding sites for these factors are not over-represented in promoters for the CTDGs. Mutational analyses of the HIV-1 LTR have demonstrated that this set of transcription factor binding sites is especially suited to confer a high response to the Cyclin T1/p-TEFb complex when it is recruited to the viral LTR via Tat and TAR RNA [64]–[67]. However, we found here that many cellular genes which are dependent upon Cyclin T1 for their up-regulation in activated CD4^+^ T cells and macrophages do not possess the combination of transcription factor binding sites that is found in the HIV-1 LTR. Thus, the combination of binding sites for NF- κB, Sp1, and the TATA is not essential, and may not be common, for CTDGs in activated CD4+ T cells and macrophages. It should be noted, however, that our study did not investigate whether the set of CTDGs are stimulated directly, rather than indirectly, by Cyclin T1.

Binding sites for the transcription factors SREBP1 and ARP1 are over-represented (p value<0.00013 and <0.0003, respectively) in the CTDGs in this study, as the promoters for 21 of 54 genes contain at least one predicted binding site for these factors (Fig. 3). SREBP1 is a membrane-associated protein that is activated by limited proteolysis and transported to the nucleus where it regulates genes involved in lipid and cholesterol metabolism [68]. ARP1 (also termed COUP-TFII) regulates metabolic homeostasis and can act as both an activator or repressor of target genes [69]. The 21 CTDGs whose promoters contain binding sites for SREBP1 and/or ARP1 may be involved in regulating metabolic conditions in both CD4^+^ T cells and macrophages, and this may be important for HIV-1 replication. This raises the possibility that variation in metabolism between HIV-infected individuals may be a contributing factor to differences in the course of disease.

### Potential for new therapeutic targets

Our initial experiments to examine a role of CSNK1G1 in HIV-1 replication suggests that it functions to enhance virion infectivity, as a siRNA depletion appeared to reduce infectivity (Fig. 5) and a dn-CSNK1G1 protein inhibited Gag processing (Fig. 6). Additional work is required to elucidate mechanisms whereby CSNK1G1 affects virion infectivity and whether this host protein has the possibility to serve as a target for anti-viral therapeutics. Although several CTDGs identified in this study play important roles in HIV-1 infection, in most cases the encoded proteins are unlikely to be targeted by HIV-1 proteins through direct protein-protein interactions. Many of these cellular proteins may regulate general cellular processes, and other proteins involved in these processes could be the direct targets of viral proteins. For example, Tat function is sensitive to the depletion of CDK11 (Fig. 4). Rather than having a direct effect on Tat function, CDK11 may regulate the level of CDK9 protein expression or the processing of HIV-1 transcripts, and because P-TEFb function and splicing are linked [15], the depletion of CDK11 may have an especially inhibitory effect on viral transcripts activated by Tat. HIV-1 co-factors that regulate general cellular processes are unlikely to be feasible therapeutics targets, as the actions of small molecules that interfere with their function are likely to be cytotoxic.

However, a number of cellular co-factors that mediate HIV-1 replication are feasible candidates for anti-viral drugs. Some co-factors in the 54 CTDGs identified here, and in the 273 factors identified in the recent large-scale siRNA screen [48], are likely to exist in multiprotein complexes that are targeted directly by viral proteins. The future characterization of these complexes and their role in the HIV-1 life cycle can identify proteins that are direct and essential targets of viral proteins. Such proteins are feasible therapeutic targets, as small molecules can be developed that disrupt protein-protein interactions [70]. Indeed, the recently licensed drug Maraviroc is a small molecule that blocks HIV-1 entry by binding to CCR5 on the surface of cells and thereby prevents a productive interaction between CCR5 and the HIV-1 Envelope protein [71].

## Materials and Methods

### Cell culture and reagents

Mono-Mac-6 cells (MM6) were obtained from Dr. Jorge Benach (State University of New York at Stony Brook). MM6 cells were maintained in RPMI 1640 medium (Invitrogen) supplemented with 10% heat-inactivated fetal bovine serum (FBS, Hyclone), antibiotics, non-essential amino acids (Invitrogen), l-glutamine (Invitrogen), and OPI media supplement (Sigma) containing 0.15 mg of oxalacetate, 0.5 g of pyruvate and 8.2 mg of bovine insulin. Jurkat cells were purchased from ATCC and maintained in RPMI 1640 medium supplemented with 10% FBS and antibiotics. For LPS treatment of MM6 cells, cultures were treated at a final concentration of 1 ng/ml lipopolysacchride (LPS, Sigma); for PMA treatment of MM6 cells, cultures were treated at a final concentration of 10 ng/ml phorbol 12-myristate 13-acetate (PMA, Sigma). For PMA+ ionomycin treatment of Jurkat cells, cultures cells treated at a final concentration of 1 ng/ml PMA (Sigma) and 1 µM ionomycin. HeLa cells and 293T cells were maintained in 10% FBS and antibiotics.

### Cell Extracts and Immunoblots

Cell extracts were prepared by incubating cells in lysis buffer (50 mM Tris, 120 mM NaCl, 0.5% NP-40) containing protease inhibitors (2 µg/ml aprotinin, 1 µg/ml leupeptin, 2.5 mM phenylmethylsulfonyl fluoride) as described previously [72]. Protein concentrations were determined by a Bio-Rad protein assay, and 20 µg of total protein was loaded onto sodium dodecyl sulfate-9% polyacrylamide gels. The procedure for immunoblots using enhanced chemiluminescence for detection has been described previously [73]. Antibody to β-actin was purchased from Sigma, and other antibodies were purchased from Santa Cruz Biotechnology.

### ShRNAs, siRNAs, and lentiviral vector production

The target sequences used for this study were: shRNA-CycT1, GCAGCGTCTTAACGTCTCA; shRNA-Control (MM), GCTATAGCTGTTCTAGTTC; CDK11, CAUGAGUAUUUCCGCGAGA; CSNK1G1, GACCGAACAUUUACUUUGAUU. The siRNAs were purchased from Dharmacon. Construction of the viral vector has been described previously [30]. The FG12 lentiviral vector is a self-inactivated lentiviral vector carrying an eGFP expression-cassette; the vector does not encode any viral gene products [35].

Stocks of the FG12 lentiviral vectors pseudotyped with vesicular stomatitis virus (VSV)-G were produced by calcium phosphate-mediated transient transfection of HEK-293T cells. Briefly, HEK-293T cells were cultured in DMEM (GIBCO Invitrogen) containing 10% FBS (HyClone), and antibiotics. The cells were cotransfected with 5 ug of: shRNA vector plasmid, the HIV-1 lentiviral packaging constructs pRSV/REV and pMDLg/pRRE, and the VSV-G expression plasmid pHCMV-G. Virus stocks were collected from the culture supernatants on days two post-transfection and were titered on HEK-293T cells based on GFP expression. MM6 cells (2×10^5^/ml) were transduced at a multiplicity of infection (m.o.i.) of five in the presence of 5 ng/ml polybrene (Sigma).

### Microarray analysis

The primary microarray data was deposited at the NCBI GEO data base (accession numbers GSE10232, GSE10233, GSE10234). Microarray analysis was performed by the Baylor Microarray Core Facility (Baylor College of Medicine, Houston, TX 77030, USA). Detailed protocols can be found at the website: http://public.bcm.tmc.edu/mcfweb/index.htm. Briefly, RNA was isolated using the Qiagen RNeasy kit according to the manufacturer's protocol and RNA quality was determined using an Agilent 2100 Bioanalyzer. RNA was reverse transcribed to generate cDNA and transcribed using T7 RNA polymerase and biotinylated ribonucleotides to generate labeled cRNA. Fragmented cRNA was hybridized to U133 plus 2.0 human gene chips (Affymetrix) containing nearly 55,000 probe sets representing over 18,953 transcripts. Following washing and staining, the arrays were scanned using an Affymetrix Gene Chip Scanner 3000. For all experiments, the 5′/3′ ratios of GAPDH ranged between 0.85 and 0.91. Comparisons of matched control and were performed for each of three independent experiments. Microarray data was analyzed with online software from Genesifter.

To generate random lists of genes whose mRNAs are expressed in both MM6 and Jurkat cells, microarray data were normalized by calculation of the GC Robust Multi-Array Average (GCRMA); random gene lists were then generated from genes that were expressed in both cell lines at log_2_ expression levels >3. To determine if genes in lists contain a published link to HIV-1 replication, gene names (and aliases) were searched in the NCBI Pub Med Database for a link to HIV. Genes were also analyzed in the NCBI HIV-1 Human Protein Interaction Database.

### Real-time RT-PCR analysis

Quantitative real-time RT-PCR was performed using the Bio-Rad MyIQ single color detection system. Cellular RNA was used to perform cDNA synthesis using the iScript cDNA synthesis kit (Bio-Rad). Briefly, 1 µg of RNA was reverse transcribed in a 20-µl reaction volume using the manufacturer's protocol. PCR reactions were performed using 3 µl of cDNA in a 50 µl reaction containing 25 µl of 2× iQ SYBR Green Supermix (Bio-Rad) and 200 nM final concentration of each primer. PCR reactions were carried out in 96-well format using a Bio-Rad iCycler with a 3 min hot start followed by 40 cycles of 15 s at 95°C, 1 min annealing and amplification at 55°C. Analysis was performed using the MyIQ software program (Bio-Rad). The threshold crossing (Ct) value for each reaction was determined and the fold-change (ΔΔCt value) was calculated with the following formulas using GAPDH as a reference control:

Primers for quantitative PCR were designed using Beacon Designer 2.0 (Premier Biosoft). All primer pairs produced single amplification products as determined by gel electrophoresis as well as melt-curve analysis using the MyIQ system. Primers used: APOBEC3F (forward): CCGCAGGCAGGGAACAAGG, APOBEC3F (reverse): AGGCATCCATTCACCAGGCATC; CX3CR1(forward): AGAGGCTGGTTCTTACGATGGC, CX3CR1(reverse): GGAGAGTTGGGTTACGGAAGGC; GAPDH (forward) CGCCAGCCGAGCCACATC, GAPDH (reverse) AATCCGTTGACTCCGACCTTCAC; HAVCR2 (forward): TGCTGCTGCTGCTGCTACTAC, HAVCR2 (reverse): GACCGACCTCCGCTCTGTATTC; HLADR1(forward): TTTGATGCTCCAAGCCCTCTCC, HLADR1(reverse): GATGCCCACCAGACCCACAG; MARCKS(forward): CGCCCAGCAACGAGACCC, MARCKS(reverse): CCTCAGCCTCACCGCCTTC; PTGS1 (forward): TTATGGCTGCTGGGCTGAGTG, PTGS1 (reverse): GCTTCCTGTCCTTCCTGCTCTG; TDO2(forward): AACCTCCGTGCTTCTCAGACAG, SPP1(forward): CAGGAGGAGGCAGAGCACAG; VEGF (forward): GCGTGCGAGCAGCGAAAG, VEGF (reverse): GGCGGTGTCTGTCTGTCTGTC, VPS24 (forward): AAAGCCCAGCCACTGTCTCAC, VPS24 (reverse): CCACCGCACCCAGCCAAG.

### HIV-1 reporter virus experiments and Gag processing experiments

For reporter virus experiments in CDK11 depletion experiments, a HeLa cell culture was infected with lentiviral DHFR-Renilla Luciferase virus containing an eGFP marker protein. Infected cells were sorted for eGFP expression, and were then infected with either HIV-1 NL4-3-Firefly Luciferase virus (wild type Tat) or with HIV-1 NL4-3 Firefly Luciferase virus encoding a mutant Tat [27]. The infected cultures were then transfected with 50 pmol of siRNAs against CDK11 or Control siRNAs. Cell extracts were prepared 48 h post-transfection and assayed for firefly and renilla Luciferase expression.

For siRNA depletions of CSNK1G1, 293T cells in 12-well culture dishes were transfected with 120 pmol of either siRNAs against CSNK1G1 or negative control siRNAs (Cyclin T1 mismatch control siRNAs). One day after siRNA transfections, cells were co-transfected with HIV-1 proviral plasmid pNL4-3-Luciferase and a plasmid expressing the VSV G glycoprotein (pVSV-G). Two days after plasmid co-transfections supernatants were collected and p24 amounts were measured by ELISA (Beckman-Coulter). HIV-1 infectivity in supernatants was measured by infecting HeLa cells with equal volumes of virion-containing supernatants, Luciferase expression was measured two days post-infection, and Luciferase expression was normalized to p24 amounts in supernatants.

The Quikchange site-directed mutagenesis kit (Stratagene) was used to introduce the K73R mutation into a CSNK1G1 cDNA. Wild-type and mutant CSNK1G1 cDNA were cloned into a C-terminal HA-tagged pcDNA 3.0 plasmid (Invitrogen). To examine Gag processing, 293T cells were transfected with Lipofectamine 2000 (Invitrogen) at Day 0 with the CSNK1G1 constructs or empty vector (pcDNA3.0). On day 1, cells were transfected with pNL4-3.GFP proviral reporter. On day 2, supernatants were centrifuged at 1,000×g for 10 minutes, and lysed with 0.5% Triton X-100 in PBS; cells were lysed with 1× Cell Culture Lysis Reagent (Promega). Equal volumes of supernatant and 40 ug of cell lysate protein were heated at 95°C for 10 minutes, run on 10% polyacrylamide gels, and transferred to nitrocellulose membranes. Blocking with 5% non-fat dried milk in TBS-T (50 mm Tris pH 8.0, 200 mM NaCl, 0.2% Tween-20) was performed for 1 hour, and primary antibody (p24 monoclonal, NIH AIDS Research and Reagent Program;183-H12-5C from Dr. Bruce Chesebro and Kathy Wehrly) binding was done overnight in the same solution at a dilution of 1∶1000. Horseradish peroxidase-conjugated secondary antibodies (Santa Cruz Biotechnology) were bound for 1 hour in 0.5% non-fat dried milk in TBS-T and SuperSignal West Pico Substrate (Pierce) was used for detection. Bands were quantified by scanning the film with a Molecular Dynamics Densitometer and quantifying bands with Image Quant 5.2 software (Molecular Dynamics).

## Supporting Information

Figure S1 A. LPS-treated MM6 cells. MM6 cells were infected with the shRNA-T1 or shRNA-Con (Cyclin T1 mismatch) lentiviral vector for five days, treated with LPS for 24 hours, and total RNA was isolated. Quantitative real-time RT-PCR assays were used to measure the expression levels of the indicated mRNAs. The fold-change value represents the change in mRNA levels in cells relative to parental MM6 cells after normalization to GAPDH levels. Fold-change values after LPS treatment as measured in microarray hybridizations were: MARCKS, 0.30; TDO2, 0.06; SPP1, 0.45; HLA-DR, 0.50; CX3CR1, 0.12. B. PMA/ionomycin-treated Jurkat cells. Jurkat cells were infected with the shRNA-T1 or shRNA-Con (Cyclin T1 mismatch) lentiviral vector for five days, treated with PMA/ionomycin for 24 hours, and total RNA was isolated. Quantitative real-time RT-PCR assays were used to measure the expression levels of the indicated mRNAs. The fold-change value represents the change in mRNA levels in shRNA-T1-infected cells relative to shRNA-Con-infected cells after normalization to GAPDH levels. Fold-change values after PMA-ionomycin treatment as measured in microarray hybridizations were: APOBEC3F, 0.26; PTGS1, 0.20; CHMP4, 0.32; VEGF, 0.22; HAVCR2, 0.26.
(0.06 MB TIF)

Figure S2 PMA-treated and LPS-treated MM6 cells have distinct RNA expression profiles. A dendrogram was constructed with the data from all probe sets for all 15 microarrays used to analyze parental, PMA-treated, or LPS-treated MM6 cells (uninfected, shRNA-T1-infected, shRNA-Con (Cyclin T1 mismatch)-infected). The Pearson Correlation distance was calculated to represent the expression differences between the microarrays. The leaves of the tree represent each of the 15 microarrays used in this study. The branches denote the relative distances between the samples. Branch joints near the leaves of the tree represent high similarity, while deeper joints represent less similarity.
(0.06 MB TIF)

Figure S3 PMA/ionomycin-treated Jurkat cells depleted for Cyclin T1 have a distinct gene profile from PMA/ionomycin-treated control Jurkat cells. A dendrogram was constructed based on the data from all probe sets for all 8 microarrays used with Jurkat cells. The Pearson Correlation distance was calculated to represent the expression differences between the microarrays. The leaves of the tree represent each of the 8 arrays used. The branches denote the relative distances between the samples.
(0.04 MB TIF)

Figure S4 Gene Ontology Analysis of CTDGs. A GO content analysis of the gene list in Table 1 was performed by tabulating the gene list against the GO structure. The color of each GO term corresponds to its adjusted P-value, with color intensity indicated by color scaling.
(0.06 MB TIF)
