# Supplementary figures and images for: Cyclin T1-Dependent Genes in Activated CD4+ T and Macrophage Cell Lines Appear Enriched in HIV-1 Co-Factors
Source: PLoS One. 2008 Sep 5;3(9):e3146. doi: 10.1371/journal.pone.0003146 (PMC2519787; doi:10.1371/journal.pone.0003146)

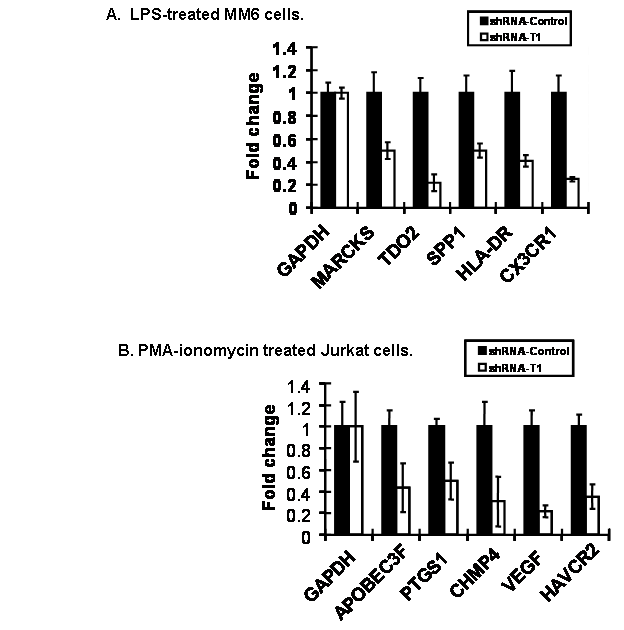

Supplement: Figure S1 — A. LPS-treated MM6 cells. MM6 cells were infected with the shRNA-T1 or shRNA-Con (Cyclin T1 mismatch) lentiviral vector for five days, treated with LPS for 24 hours, and total RNA was isolated. Quantitative real-time RT-PCR assays were used to measure the expression levels of the indicated mRNAs. The fold-change value represents the change in mRNA levels in cells relative to parental MM6 cells after normalization to GAPDH levels. Fold-change values after LPS treatment as measured in microarray hybridizations were: MARCKS, 0.30; TDO2, 0.06; SPP1, 0.45; HLA-DR, 0.50; CX3CR1, 0.12. B. PMA/ionomycin-treated Jurkat cells. Jurkat cells were infected with the shRNA-T1 or shRNA-Con (Cyclin T1 mismatch) lentiviral vector for five days, treated with PMA/ionomycin for 24 hours, and total RNA was isolated. Quantitative real-time RT-PCR assays were used to measure the expression levels of the indicated mRNAs. The fold-change value represents the change in mRNA levels in shRNA-T1-infected cells relative to shRNA-Con-infected cells after normalization to GAPDH levels. Fold-change values after PMA-ionomycin treatment as measured in microarray hybridizations were: APOBEC3F, 0.26; PTGS1, 0.20; CHMP4, 0.32; VEGF, 0.22; HAVCR2, 0.26. (0.06 MB TIF) [file pone.0003146.s001.tif]

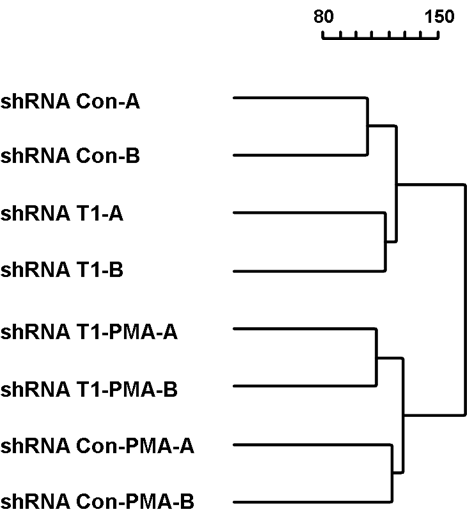

Supplement: Figure S3 — PMA/ionomycin-treated Jurkat cells depleted for Cyclin T1 have a distinct gene profile from PMA/ionomycin-treated control Jurkat cells. A dendrogram was constructed based on the data from all probe sets for all 8 microarrays used with Jurkat cells. The Pearson Correlation distance was calculated to represent the expression differences between the microarrays. The leaves of the tree represent each of the 8 arrays used. The branches denote the relative distances between the samples. (0.04 MB TIF) [file pone.0003146.s003.tif]

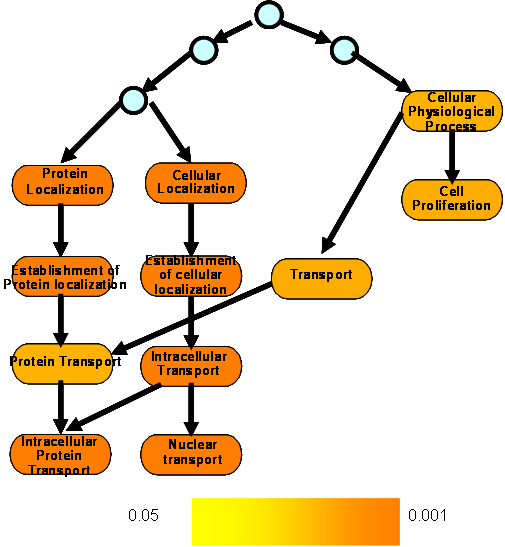

Supplement: Figure S4 — Gene Ontology Analysis of CTDGs. A GO content analysis of the gene list in Table 1 was performed by tabulating the gene list against the GO structure. The color of each GO term corresponds to its adjusted P-value, with color intensity indicated by color scaling. (0.06 MB TIF) [file pone.0003146.s004.tif]
